# Supplementary material for: BBQ methods: streamlined workflows for bacterial burden quantification in infected cells by confocal microscopy
Source: Biol Open. 2024 Jan 22;13(1):bio060189. doi: 10.1242/bio.060189 (PMC10836645; doi:10.1242/bio.060189)
Supplement: Supplementary information [file biolopen-13-060189-s1.pdf]

Table S1.

Oligonucleotides name	Oligonucleotides sequence (5'-3')	Note
cat-P*tet* -yfp-P1	aaaccccagccacagaaataattcggtttatccccgctggcgcggggaacacgctcatgtAATGGCGCGCCTTACGCCCC	For S.Tm cat-*Ptet*- yfp chromosomal integration
cat-P*tet* -yfp-P2	aaaccggcgaggtcaataaaaaatggtgtggctttaccgtgttccccgcgccagcgggaTGGAAGAAATAGCGCTTTCAGC	
S-insert-verify-F	AACCGCATAGCCTCTTTCG	For verifiying cat-*Ptet*- yfp integration
S-insert-verify-R	CGCTCGAATTTATTTGAGGC	
m-TGA-y-*cadBA* -IF	cttcacctcgattcgaatgtgaaaaacatgttgccaaaa	For *cadBA* transcriptional fusion
m-TGA-y-*cadBA* -IR	aaaattattggaattgtcgactcccgctccccatcatat	
m-TGA-y-*hmpA* -IF	cttcacctcgattcgaatcttcctgacgtactttttcctgc	For *hmpA* transcriptional fusion
m-TGA-y-*hmpA* -IR	tattggaattgtcgactcagtttcggtcctgtttcaacc	
m-TGA-y-*katG* -IF	cttcacctcgattcgaaccacttctacacgttgaatcgtgc	For *katG* transcriptional fusion
m-TGA-y-*katG* -IR	tattggaattgtcgaccctgatggaaaggacattttccag	

Dataset 1.

Available for download at
https://journals.biologists.com/bio/article-lookup/doi/10.1242/bio.060189#supplementary-data

Biology Open • Supplementary information
